# The fruits of our labour: Interpersonal coordination generates commitment by signalling a willingness to adapt

**DOI:** 10.1177/17470218221079830

**Published:** 2022-03-03

**Authors:** Luke McEllin, Annalena Felber, John Michael

**Affiliations:** 1 Department of Cognitive Science, Central European University, Budapest, Hungary; 2 Department of Psychology, University of Warwick, Coventry, UK

**Keywords:** Coordination, decision-making, adaptation, commitment, cooperation

## Abstract

Countless everyday activities require us to coordinate our actions and decisions with others. Coordination not only enables us to achieve instrumental goals, but has also been shown to boost *commitment*, leading people to persevere with an interaction even when their motivation wavers. So far, little is known about the mechanism by which coordination generates commitment. To investigate this, we conducted two experiments that represented very different coordination problems: coordination of movement timing on a joint drumming task (Experiment 1) and coordination of decision-making on a joint object matching task (Experiment 2). In both experiments, the similarity of the participant and partner was manipulated by varying whether or not they had perceptual access to the participant’s workspace, and the participants’ attribution of (un)willingness to invest effort into the joint action by adapting was manipulated by varying whether or not the participant believed their partner had perceptual access. As a measure of commitment, we registered how much participants’ persisted on a boring and effortful task to earn points for their partners. Participants were significantly less committed to earning points for unadaptive partners than for adaptive partners, but only when they believed that their partner was unwilling to adapt rather than unable to adapt. This demonstrates that coordination can generate commitment insofar as it provides a cue that one’s partner is willing to invest effort to adapt for the good of the interaction. Moreover, we demonstrate that this effect generalises across different kinds of coordination.

## Introduction

Coordination is ubiquitous throughout our day-to-day lives. Whether we are assembling vehicles, directing movies, or performing open-heart surgery, we coordinate actions and decisions to achieve our goals effectively. Moreover, there is a wealth of research showing that coordination generates important indirect benefits by boosting prosocial attitudes and motivations: strengthening social bonds (Reddish, 2012), enhancing trust and rapport (Hove & Risen, 2009; Launay et al., 2013), and increasing cooperation and helping (Reddish et al., 2013; Wiltermuth & Heath, 2009). Indeed, coordination has also been shown to yield other social effects, modulating memory of self and other, and even increasing imitation of a co-actor (Cross et al., 2021).

Building on this research, interpersonal coordination has also been shown to generate a sense of *commitment* leading people to persist longer on a boring or effortful task to benefit another agent (Michael et al., 2016a). This finding is particularly important insofar as commitment serves as a glue holding human social life together (Michael et al., 2016b). Commitment is crucial not only in sustaining small-scale social interactions unfolding over brief timescales, but also in providing the stability and predictability required for large-scale collective actions, such as combating climate change or pandemics, which involve sustained effort and personal sacrifice over longer timescales.

In a coordinated interaction, which cues contribute to the development of a sense of commitment towards a co-actor? One cue that generates commitment is likely the similarity between each other’s actions and decisions. With regards to the prosocial consequences of coordination, several mechanisms have been proposed which are sensitive to the similarity of movement timing when coordinating, with these potential mechanisms involving inferences on the basis of the similarity between actors’ behaviour. For instance, it has been suggested that similarity between movement timing may cue inferences about higher level conceptual similarity, such as similarity between goals and intentions, or even at the level of traits or psychological states (Miles et al., 2009; Reddish et al., 2013). Alternatively, similarity at the level of movement timing has been suggested to create a merging between self and other (Hove & Risen, 2009) and to de-individuate actors, thereby increasing their awareness of their role as interdependent units of that group (Cross et al., 2019).

A less explored possibility is that being involved in a coordinated interaction may provide an actor with cues to a partner’s willingness to invest effort into the interaction, boosting an agent’s sense of commitment towards that partner. This is motivated by the observation that successful coordination requires agents to invest effort to adapt to each other, thereby making their actions or decisions easier for their partners to align with (Bardsley et al., 2010; Keller et al., 2014). In other words, adaptation reflects an agent’s willingness to invest effort into an interaction, insofar as it requires an agent to incur an individual cost (e.g., biomechanical or cognitive) to reduce the (e.g., planning or anticipation) costs for their partner and/or to increase the chances of jointly succeeding (Török et al., 2019). For this reason, an agent’s willingness to adapt may create a sense of debt or obligation towards her, making one feel committed to “repay one’s debt” to her (McGrath & Gerber, 2019). This hypothesis is consistent with recent research demonstrating that when an agent invests effort in a joint action, this increases her partner’s sense of commitment towards the joint action and towards her, leading that partner to persist longer on boring or effortful tasks (Chennells & Michael, 2018; Székely & Michael, 2018).

## The current research

In most instances of coordination, we cannot tease apart effortful adaptation from similarity: by adapting to one another, two agents increase the similarity between their actions or decisions. To do this, we aimed to create a task in which we should make different predictions depending on whether the mechanism by which coordination generates commitment involves inferences about similarity only, or inferences about a partner’s willingness to invest effort. Specifically, a mechanism that is sensitive only to the similarity between one’s own and one’s partner’s actions would not be sensitive to a partner’s willingness to invest effort and would therefore not discriminate between behavioural similarity and the intention to adapt one’s movements to facilitate coordination. However, a mechanism that is sensitive to a partner’s willingness to invest effort into the interaction would be sensitive to whether or not one’s partner is wilfully adapting their behaviour for the good of the actor and the interaction. This would also mean that any inferences being made based on similarity or dissimilarity would be moderated by beliefs about whether one’s partner was or was not willingly investing an adequate amount of effort to achieve behavioural similarity. To tease apart similarity and willingness to invest effort, we manipulated two factors separately. First, we manipulated whether the participant interacted with a partner who was adaptive, and who therefore exhibited similar actions and decisions to the participant, or a partner who was unadaptive, and who therefore exhibited dissimilar actions and decisions to the participant. Second, we manipulated whether participants were led to believe that their partner was able or unable to adapt to them, and consequently, what inferences they were likely to draw from the interaction about their partner’s willingness to invest the effort required to adapt. We reasoned that by leading participants to believe that their partner was unable to adapt, we would lead them to attribute the unadaptive partner’s lack of adaptivity to an *inability to adapt* (unable-to-adapt condition). In contrast, we expected that by leading participants to believe that their partner was able to adapt, we would lead them to attribute the unadaptive partner’s lack of adaptivity to an *unwillingness to invest the effort required to adapt* (able-to-adapt condition).

To provide a general test of our hypotheses, we devised two separate experimental scenarios implementing two distinct forms of coordination: action coordination (Experiment 1) and decision-making coordination (Experiment 2). This allowed us to examine whether any effects we may find would generalise across different coordination problems, in which adaptation require different kinds of effort investment (e.g., physical vs. mental effort) and yield different kinds of behavioural similarity (e.g., perceptual similarity vs. abstract similarity).

## Experiment 1

In Experiment 1, we instructed participants to coordinate drum taps with one adaptive partner and one unadaptive partner, also manipulating whether or not the participant believed that these partners were unable to adapt or unwilling to adapt. As an index of the participant’s level of commitment to each of these partners, we measured how long they persisted on a task in which they needed to tap a spacebar to charge a battery to earn points for each of the partners.

When coordinating, if participants are sensitive only to the similarity between their own and their partner’s movement timing, then we should expect that participants would charge the batteries more (thereby accruing more points) for the adaptive partner than for the unadaptive partner, irrespective of their beliefs about whether or not their partners were in fact able to adapt. However, if participants are also sensitive to their partner’s willingness to invest effort by adapting their movement timing, we should expect that participants would charge the batteries more for the adaptive partner than the unadaptive partner, but only when they believed that their partners had the ability to adapt (i.e., in the able-to-adapt condition), thus attributed the unadaptive partner’s lack of adaptivity to an unwillingness to adapt.

### Experiment 1: method

The hypotheses, sample sizes, methods, and initial analyses were all pre-registered (after piloting) before data collection. The pre-registration can be accessed at: https://aspredicted.org/blind.php?x=bm5dj5

### Participants

Using an online participant recruitment system (SONA: Central European University), we recruited 28 participants for the able-to-adapt condition (*M* = 23.4, *SD* = 3.6, 15 females) and 28 participants for the unable-to-adapt condition (*M* = 24.5, *SD* = 2.9, 11 females). A g*power analysis suggested that we recruit 27 participants in each group, to provide 95% statistical power for detecting a medium effect size (η_p_^2^ = .06), however, we decided to collect 28 in each group to ensure equal counterbalancing. This study was approved by the United Ethical Review Committee for Research in Psychology (EPKEB). The study was performed in accordance with the Declaration of Helsinki.

### Apparatus and stimuli

**Figure 1.**
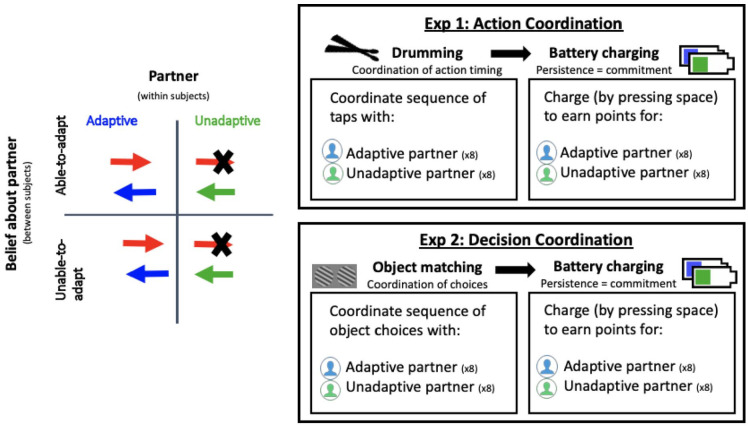
The left panel depicts the design of the two experiments. The arrows represent the direction of information flow in the two experiments (Experiment 1: tapping sounds and Experiment 2: visual access to partners’ workspace). The red arrow represents the participant, the blue arrow represents the adaptive partner, and the green arrow represents the unadaptive partner. The right panel depicts the structure of both experiments.

Figure 1 provides a schematic of the experimental setup. The participant and the confederate sat in adjacent but connected rooms. Participants tapped mounted drum pads in synchrony, with instructions and a metronome being presented through a desktop computer. They then completed a battery-charging task, by tapping spacebar of the computer to fill the battery with power.

#### Drumming task

In each room, the participant and confederate tapped mounted DDRUM drum pads with a standard drumstick (40 cm × 1.5 cm). The MIDI input from the drum pads was sent to a PC via a DDTI trigger box, which allowed us to control the sensitivity of the drum pads, as well as the tone they triggered. We also used the box to exclude double taps (taps that occurred less than 100 ms between each other), as these were due to the drumstick vibrating on the drum pad being registered as extra taps. The MIDI output was sent to Roland headphones via a Focusrite audio interface. Metronome playback and MIDI recording was done in Ableton Live and Python, and stimuli and instructions were presented on PsychoPy2 (Python 2.7).

#### Battery-charging task

The battery-charging task was implemented with a custom programme using PsychoPy2 (Python 2.7). The programme displayed a battery (350 × 800 pixels), and the avatar representing the partner (100 pixels) on the screen. A bar (maximum of 250 × 600 pixels) representing the battery’s level of power was displayed on the left edge of the battery and would gradually increase in length (6 pixels, every time the number of presses passed a threshold of *x*^3^/*y*, where *x* is the number of 6-pixel units displayed in the battery and *y* is the number of presses) as participants tapped the space bar, creating the appearance of the battery filling with power. As the amount of power in the battery increased, the number of taps required to fill the battery with more power would also increase, making the task progressively more difficult. See Supplementary Material 1 for a screenshot of the battery.

#### Instructions

Instructions and the trial structure were all presented on PsychoPy2. At the start of every trial, the trial and block number were displayed, and then, once participants hit the drum, the instructions for that trial were displayed on the screen.

### Procedure

After providing their informed written consent, participants were informed that they had been randomly selected to play the role of the red player, and would be participating in a two-part experiment, sometimes paired with a blue player, and sometimes paired with a green player. We told participants that they would be seated in separate rooms to their two partners to preserve anonymity, which allowed us also to control for communication and observable cues to similarity (e.g., gender, clothing, body size), meaning that the only dimension in which participants could be similar or different was their performance on the task. Once they had read the instructions, participants were asked to complete a “rhythm test.” For this rhythm test, they were instructed to tap at a steady pace, and informed that any participants who received a score below a certain threshold would be excluded from participating in the experiment. This was done to ensure that participants believed that their partners were competent, and thus that they would not attribute their partners’ lack of adaptivity to a lack of rhythmic ability. After the rhythm test, we reiterated the instructions and asked participants to complete two practice trials (one with each partner).

First, participants completed a *synchrony task*, which required them to synchronise 32 drum taps with either the “blue partner” or the “green partner.” Both roles were in fact played by the same confederate: this minor form of deception was necessary to maintain experimental control, as our manipulations precisely targeted the adaptivity of the partners and participants’ beliefs about the reasons for that adaptivity (or lack thereof). For every trial, to ensure that adaptation was required for effective coordination (i.e., both actors started out unsynchronised), both actors would listen to and tap along to differently paced metronomes (96 or 120 BPM counterbalanced across trials) for eight beats before they started attempting to synchronise with their partner. We ensured that one of these partners would be adaptive simply by allowing the participant and the confederate to hear each other, meaning that they could adapt to each other, thus resulting in similar movement timing. We ensured that the other partner would be unadaptive by preventing the confederate from hearing the participant; this made it impossible to adapt to the participant, resulting in dissimilar movement timing. Participants completed eight trials with the adaptive partner, and then eight trials with the unadaptive partner. Which of the two (i.e., the blue or the green partner) was adaptive, and which was unadaptive, and the order in which participants synchronised with the adaptive and unadaptive partner was counterbalanced across participants. Between these blocks, participants were instructed to take a short break (~10 min) while they believed that the other participants were completing the task together.

Participants were randomly assigned either to the *able-to-adapt belief* condition, *or unable-to-adapt belief* condition. In the unable-to-adapt belief condition, participants were led to believe that both of their partners could only hear themselves. This was to ensure that they would attribute their “unadaptive” partner’s lack of adaptivity to the fact that their partner could not hear them, and thus refrain from drawing any negative inferences about that partner’s willingness to adapt. In the able-to-adapt belief condition, participants were led to believe that both partners could hear them (the participant). This was to ensure that they would attribute the “unadaptive” partner’s lack of adaptivity to an unwillingness to invest effort in the interaction by adapting. Participants were not incentivised during the synchrony task; they did not receive any reward for synchronising.

Next, participants completed the *battery-charging task*, in which they were instructed to charge on-screen batteries (see to earn points for the partner). They charged the battery by tapping the spacebar for as long as they were willing (up to a maximum of 1,000 space bar taps), pressing escape when they wanted to stop charging. They were instructed that the more they charged the battery, the more points they would accrue for that partner. This was completed eight times for the adaptive partner and eight times for the unadaptive partner, in an alternating order (whether participants started with the adaptive or unadaptive partner was counterbalanced across participants) with the avatar of the partner whose battery was to be charged being displayed on the screen, along with a power bar that matched the avatar’s colour.

At the end of the experiment, we administered questionnaires to measure participant’s empathy and affiliation towards their partners (the same questionnaires as those used in the work of Cross et al., 2016). We also asked participants whether they believed they were being deceived with regards to their partner (i.e., whether they believed that they were actually interacting with a real participant), to exclude any participants who believed that they were interacting with a confederate/algorithm.

### Experiment 1: results

**Figure 2.**
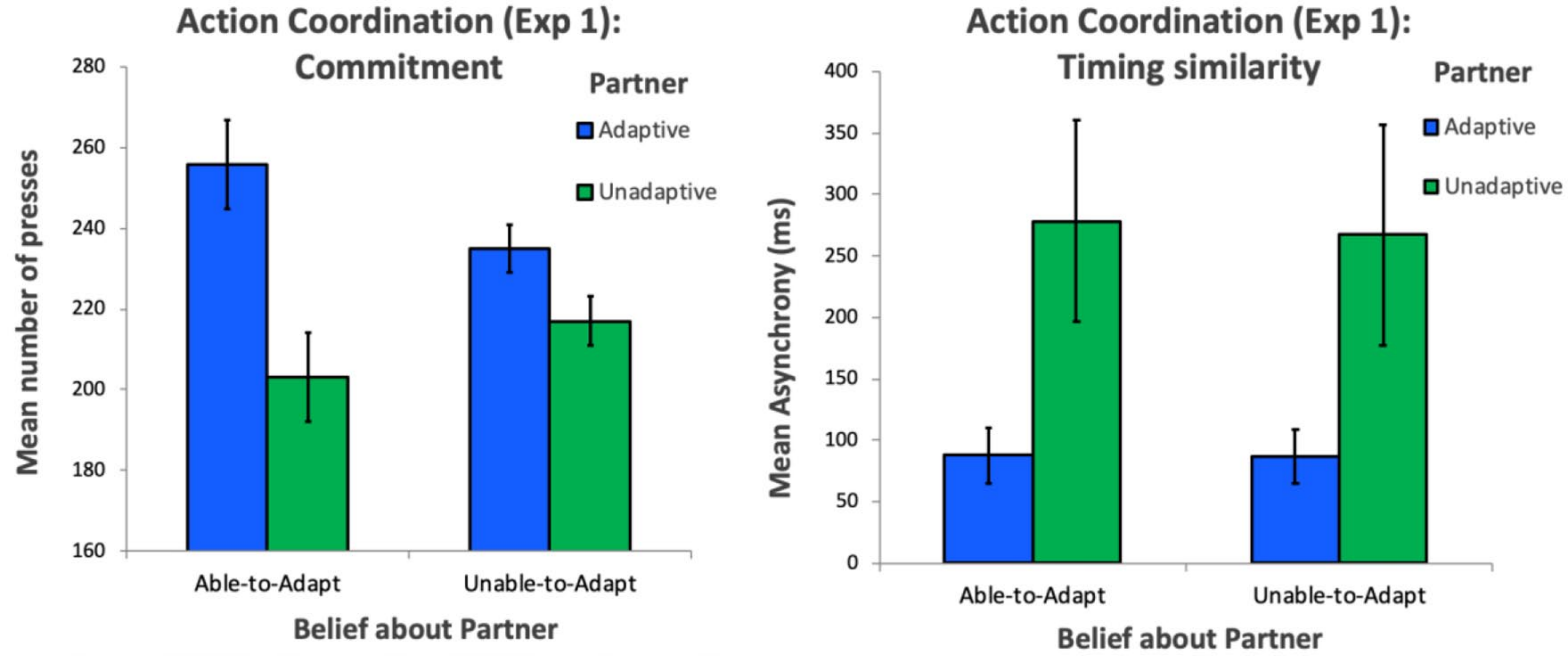
Left panel displays commitment (indexed by persistence on the charging task) to the adaptive and unadaptive partner in the able-to-adapt and unable-to-adapt belief conditions. Right panel displays movement timing similarity (indexed by drumming asynchrony) with the adaptive and unadaptive partner in the able-to-adapt and unable-to-adapt belief conditions. Error bars represent within-subject confidence intervals.

To investigate the extent to which participants invested effort—by continuously pressing spacebar—to charge batteries for the adaptive partner (whose movement timing was similar to the participants movement timing on the drumming task) and unadaptive partner (whose movement timing was different to the participant movement timing on the drumming task) in the two conditions, we carried out a 2 × 2 mixed analysis of variance (ANOVA) with mean number of spacebar presses as a dependent variable, partner (adaptive and unadaptive) as a within-subjects factor, and belief (able-to-adapt and unable-to-adapt) as a between-subjects factor. The ANOVA revealed a significant main effect of partner, *F*(1,54) = 31.41, *p* < .001, η^2^ = .34, 95% confidence intervals (CIs) (within subjects) [236.89, 255.11], [200.89, 219.11], but no main effect of belief, *F*(1,54) = .005, *p* = .94, η^2^ = .00, 95% CIs (within subjects) [217.77, 241.23], [219.52, 232.48]. Importantly, we found a significant interaction between partner and belief, *F*(1,54) = 8.00, *p* = .007, η^2^ = .09, 95% CIs (within) [244.27, 267.73], [228.52, 241.48], [191.27, 214.73], [210.52, 223.48] (see left panel of Figure 2), demonstrating that participants were relatively more committed to the adaptive partner than the unadaptive partner when they attributed lack of adaptivity down to unwillingness to adapt, rather than inability to adapt. However, post hoc *t* tests showed that participants charged the adaptive partner’s battery (mean number of spacebar presses) more than the unadaptive partner’s battery in both the partner able-to-adapt belief condition, *t*(27) = 4.75, *p* < .001, *d* = .9, and the partner unable-to-adapt belief condition, *t*(27) = 3.01, *p* = .006, *d* = .57, demonstrating that participants were overall more committed to the adaptive partner whose movement timing was more similar to their own, than the unadaptive partner whose movement timing was dissimilar to their own, regardless of their belief about whether or not the unadaptive partner was in fact able to adapt. These results show that, though similarity may play a role in the emergence of commitment irrespective of any inferences about a partner’s willingness to invest effort by adapting, a partner’s willingness to invest effort to facilitate coordination is central to the development of a sense of commitment.

Considering the nature of the battery-charging task, participants were likely to become bored and fatigued towards the end of the experiment, potentially leading to decreased persistence. Moreover, motivational differences between the conditions may have led persistence to decrease at different rates, which may help explain the mechanism by which our manipulation affected commitment. To investigate this, we carried out a linear mixed-effects model (LMM), with number of spacebar presses (for each trial) as the response variable, partner, belief and trial as fixed test effects and participant number as a random effect. We first used a likelihood ratio test to compare a full model which contained all fixed test effects with a null model containing only the random effects to ensure that our test effects explain a significant amount of the variance associated with our dependent variable (Forstmeier & Schielzeth, 2011). This analysis was significant, χ^2^ = 83.13, *p* < .001, meaning that our test effects explain a significant amount of the variance associated with the dependent variable. The final model (found using a process of backward elimination) revealed a significant effect of partner, *t* = −3.43, *p* < .001, but no effect of belief, *t* = −1.08, *p* = .28, but an interaction between partner and belief, *t* = 2.14, *p* = .032. There was a main effect of trial, *t* = −7.82, *p* < .001, but no interaction between trial and direction or knowledge, demonstrating that although motivation did decrease throughout the experiment, it did so at the same rate for all conditions.

### Controlling for similarity

To further disentangle the willingness to invest effort and the resulting similarity, we used asynchrony (timing of partner’s taps subtracted from timing of participant’s taps) in the drumming task as an index of perceptual similarity between the actor’s movements (lower asynchrony means more similarity regarding movement timing). We computed the mean asynchrony for each trial to get an index of how synchronised the participant and partner were on that trial. To get an index of coupling strength between actors, we also computed the standard deviations of the asynchrony (e.g., Mills et al., 2019). This allowed us to investigate whether actors were falling into patterns of interpersonal coupling that asynchrony would not capture (e.g., consistent leader–follower relations). Approximately, 4.5% of drumming trials were discarded—five trials were excluded due to equipment malfunction, and 32 trials were removed as outliers (>3 *SD* of the mean). However, participants did still complete these trials, meaning that they still interacted with the adaptive and unadaptive partner an equal amount of times.

First, a 2 × 2 ANOVA on the asynchrony data (mean asynchrony) yielded a significant main effect of partner *F*(1,54) = 46.54, *p* *<* .001, η^2^ = .46, 95% CIs (within subject) [65.32, 109.39], [187.1, 359.4], but no main effect of belief, *F*(1,54) = .027, *p* = .87, η^2^ = .000, 95% CIs (within subject) [130.6, 235.5], [121.37, 232.84], or no significant interaction between partner and belief, *F*(1,54) = .035, *p* = .85, η^2^ = .000, 95% CIs (within subjects) [65.2, 110,4], [65.43, 108.37], [196, 360.6], [177.3, 357.3] (see right panel of Figure 2), demonstrating that patterns of asynchrony were the same in both conditions, meaning that the differences in similarity cannot explain the differences in commitment we observed between participants in the two groups. We also observed a similar pattern of results for the standard deviation of asynchronies, namely, a main effect of partner *F*(1,54) = 23.44, *p* *<* .001, η^2^ = .3, but no main effect of belief, *F*(1,54) = .036, *p* = .85, η^2^ = .000, or no significant interaction between partner and belief, *F*(1,54) = .009, *p* = .85, η^2^ = .000, demonstrating that patterns of coupling strength (i.e., coupling strength was stronger with the adaptive partner compared with the unadaptive partner) was similar across both groups.

We also carried out an LMM, with number of space presses (for each trial) as the response variable, partner, and belief as fixed test effects adding asynchrony as a fixed control effect and participant number as a random effect. This allowed us to control for any variability associated with similarity of movement timing, allowing us to isolate the effect of adaptivity. We first compared a full model with a null model containing only the fixed control effect and the random effect, using a likelihood ratio test. This comparison was significant χ^2^ = 14.17, *p* = .003, meaning that our test effects explain a significant amount of the variance associated with the dependent variable. The final model (found using a process of backward elimination) revealed a significant effect of partner, *t* = −3.33, *p* < .001, but no effect of belief, *t* = −1.06, *p* = .292. Importantly, even with overall similarity controlled for, the interaction between partner and belief was still significant, *t* = 2.08, *p* = .037. There was no significant effect of overall asynchrony in the final model. Overall, this demonstrates that our findings cannot be explained by different patterns of movement timing similarity in the two groups, providing additional evidence that willingness to invest effort by adapting fosters commitment independently of similarity.

### Questionnaire data

Because many of the participants did not complete the questionnaires, or completed them unreliably (i.e., missing data or completely homogeneous responses indicating they did not answer the questions meaningfully), perhaps due to fatigue or demotivation after the charging task, we decided not to include them because the data would be difficult to interpret, with any conclusions from these data posing the risk of Type I or Type II error. We have included these data and the analysis in Supplementary Material 3.

## Experiment 2

Decision-making coordination, like action coordination, is pervasive in everyday life—from deciding what film to watch with one’s partner, to political parties forming strategic coalitions to enact their legislative agendas (Guala & Mittone, 2010; Schelling, 1960). In the same way that similarity regarding movement timing may lead to inferences regarding intentions, group membership or even psychological states and traits (Cross et al., 2019; Hove & Risen, 2009; Reddish et al., 2013), similarity regarding movement decisions can also foster commitment due to similar inferences. Moreover, in the same way that adaptation of movement timing may yield commitment by signalling that one is willing to invest physical effort into an interaction, adaptation of decisions can yield commitment by signalling that one is willing to invest cognitive effort into an interaction.

Experiment 2 was therefore designed to investigate whether the role of similarity and investment of effort contribute to the development of commitment towards an agent with whom we coordinate decisions.

Participants first coordinated decisions with adaptive or an unadaptive partners (while controlling for perceived willingness to adapt), before we measured commitment to these partners using the same battery-charging task as in Experiment 1. If participants are sensitive only to the similarity between decisions, we should expect participants to charge the adaptive partner’s battery more than the unadaptive partner’s battery, irrespective of their belief about whether or not their partners were willing to adapt. In contrast, the investment of effort hypothesis implies that decision-making coordination generates commitment because a partner’s willingness to tailor their decisions to ours reflects an investment of effort into the interaction. This predicts that participants will charge the adaptive partner’s batteries more than the unadaptive partner’s batteries, but only when they attributed the unadaptive partner’s lack of adaptivity to an unwillingness rather than inability to invest cognitive effort to adapt their decisions.

### Experiment 2: method

The hypotheses, sample sizes, methods, and initial analyses were all pre-registered (during very early stages of data collection). The pre-registration can be accessed at: https://aspredicted.org/blind.php?x=5ar3xj.

### Participants

We used an online system (SONA: University of Warwick) to recruit 26 participants for the able-to-adapt condition (*M* = 22.4, *SD* = 4.1, female = 15) and 26 participants for the unable-to-adapt condition (*M* = 21.3, *SD* = 2.9, female = 14). As in Experiment 1, we set out to recruit 28 participants in each group to ensure sufficient statistical power (as determined by g*power analysis) and equal counterbalancing. However, as the severe acute respiratory syndrome coronavirus 2 (SARS-CoV-2) pandemic has compelled us to close our lab for the foreseeable future, we have decided to declare data collection complete with the current sample of 52 participants. Although this resulted in unequal counterbalancing with regards to the player name assigned to the adaptive and unadaptive partners (for each group, the adaptive partner was the “blue player” 14 times and “green player” 12 times) the overall sample size, and order that the partner interacted with the adaptive and unadaptive partner was the same between the two conditions. The experiment was conducted in accordance with the Declaration of Helsinki and was approved by the Humanities & Social Sciences Research Ethics Sub-committee (HSSREC) at the University of Warwick (approval number: 01/16-17), as part of the ERC-funded project “[679092: Sense of Commitment].”

### Apparatus and stimuli

Figure 1 provides a schematic of the experimental setup. The participant first completed a decision-making coordination task with their partner (which was either an adaptive or unadaptive virtual partner), with the aim to coordinate by choosing Gabor patches (stimuli that vary on the basis of grating orientation, see Supplementary Material 2 for an example) with matching orientations. They then completed a battery-charging task, by tapping spacebar of the computer to fill the battery with power.

#### Decision-making coordination task

The participant’s task was to select a Gabor patch having the same orientation as the one chosen by their partner. Two out of the three Gabor patches in the participant’s workspace had the same orientation as two of the Gabor patches in their partner’s workspace. Thus, there were two possible matches. Participants did not see which Gabor patch their partner chose until after they had made their own decision. At this point, they received feedback in the form of a ring (25 pixels in width) around the Gabor patch that they chose and a ring around the Gabor patch chosen by their partner. On each trial, participants and their partners repeated this procedure eight times, with the aim of coordinating their choices consistently.

#### Workspace

Participants were presented with a screen displaying their own workspace on the top half of the screen, and their partner’s workspace on the bottom half of the screen. Each workspace contained an array of three Gabor patches (120 pixels) with varying orientations (0–90°), spaced (250 pixels) apart.

The partner’s workspace contained three Gabor patches with highly distinct orientations, making it easy to discriminate among all three of them. In contrast, the participant’s workspace contained one Gabor patch with a highly distinct orientation (the distinguishable patch), and two Gabor patches with similar orientations, which were therefore difficult to distinguish (two indistinguishable patches). Importantly, the oddball had the same orientation as one of the partner’s Gabor patches, and one of the two indistinguishable patches had the same orientation as one of the partner’s Gabor patches. Hence, if the partner selected the Gabor patch which matched the participant’s distinguishable patch, then the partner made coordination easy for the participant. This choice could be considered “adaptive” insofar as the decision was informed by consideration of the participant’s perspective, reflecting an investment of cognitive effort to make the task as easy as possible for the participant. This also resulted in the partner preferring similar choices to the participant (assuming the participant had a preference for the distinguishable patch). In contrast, if the partner selected the Gabor patch which corresponded to one of the participant’s two indistinguishable Gabor patches, then the partner made coordination difficult for the participant, forcing them to discriminate between two indistinguishable options. This choice could be considered “unadaptive” insofar as the decision was made without any investment of effort in considering the participant’s perspective. Moreover, this led to the partner having dissimilar preferences to the participant, with regards to which patch to choose.

#### Virtual partners

We programmed two virtual agents to act as the two partners. This minor form of deception was necessary to maintain experimental control, as our manipulations precisely targeted the adaptivity of the partners and participants’ beliefs about the reasons for that adaptivity (or lack thereof). We programmed the adaptive partner to appear considerate, choosing the Gabor patch which corresponded to the participant’s oddball patch 80% of the time, and choosing the other two options at random. This meant that throughout the trial, coordination was easy for the participant. We programmed the unadaptive partner to prefer the similar patch 80% of the time. Throughout a trial, this made coordination difficult for the participant, as they had to discriminate between two indistinguishable options. Whether participants interacted with the adaptive or unadaptive partner first, and whether the adaptive partner was assigned the colour blue or green was counterbalanced across participants.

#### Battery task

The battery-charging task was the same as in Experiment 1

### Procedure

As in Experiment 1, participants first provided informed written consent, and were escorted to separate rooms and assigned to the role of the red player. They were told they would complete a two-part experiment, sometimes paired with the blue player, and sometimes paired with the green player. After reading the instructions, participants received some additional on-screen instructions to supplement their understanding of the task.

They then started the *decision-making coordination task*, in which they were required to coordinate decisions with their partner. The participant and the partner each had a workspace containing an array of three Gabor patches with varying orientations. On each trial, the participant and the partner each selected one of their three Gabor patches. The aim was to coordinate by choosing Gabor patches with matching orientations. Importantly, the workspaces were always set up such that there were two possible matches. One of these two possible matches, however, was more difficult for the participant, because the orientation of the appropriate Gabor patch was very similar to that of one of the other two Gabor patches. For the partner, the two matches were of equal difficulty. We programmed two virtual partners, one adaptive and the other unadaptive. The adaptive partner was programmed to have a preference for the Gabor patch that was easily distinguishable for the participant, creating the impression that she was taking the participant’s perspective into consideration. In this way, the adaptive partner exhibited an investment of cognitive effort to make the task as easy as possible for the participant. Moreover, because this was the patch that the participant would be most likely to choose (as it is easiest to distinguish), this led to the participant and the partner having similar preferences thus making similar choices. We programmed the unadaptive partner to have a preference for the Gabor patch that was difficult for the participant to distinguish, creating the impression that he was making decisions without considering the participant’s perspective. Moreover, because the participant would be less likely to choose this patch (because it was difficult to distinguish), this led to the participant and the partner having dissimilar preferences thus making dissimilar choices.

Although, in a real interaction, participants may be able to solve this task with other policies (e.g., always choosing the left-most patch that occurs in one’s partners’ display), we programmed participants to have the policies described above to create clear instances of behaviour that clearly seems to either consider or ignore the participant’s perspective.

As in Experiment 1, participants were randomly assigned to either the *unable-to-adapt belief* condition or the *able-to-adapt belief* condition. In the unable-to-adapt condition, participants were led to believe that both of their partners could see only their own (i.e., the partner’s own) workspace. This was to ensure that participants in this condition would attribute the unadaptive partner’s lack of adaptivity to the fact that their partner could only see their own workspace. In the able-to-adapt condition, participants were led to believe that both partners could see both their own and the participant’s workspace. This was to ensure that participants would attribute the lack of adaptivity of the unadaptive partner to an unwillingness to invest cognitive effort in adapting their choices to facilitate the participant’s task. Participants were not incentivised during the decision-making coordination task.

In total, participants completed eight trials (eight decisions per trial) with the adaptive partner, and eight trials with the unadaptive partner. After the decision-making coordination task, participants completed the *battery-charging task*, which was the same as in Experiment 1. Between these blocks, participants were instructed to take a short break (~10 min) while they believed that the other participants were completing the task together. Due to the significant amount of missing data in Experiment 1 (likely due to fatigue and lack of motivation after completing the charging task), we decided not to administer (or pre-register) the questionnaires for Experiment 2. Although this meant that we could not investigate prosocial attitudes towards each partner, these were not necessary to interpret our objective measure of commitment.

### Experiment 2: results

**Figure 3.**
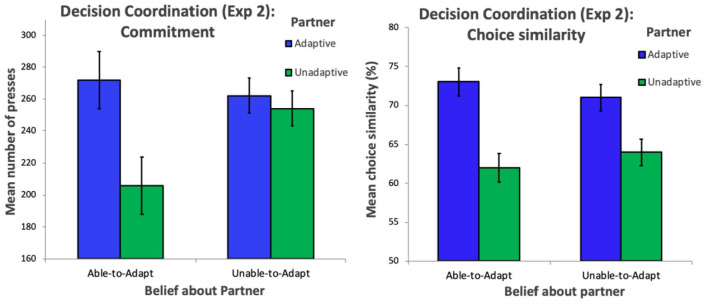
Left panel displays commitment (indexed by persistence on the charging task) to the adaptive and unadaptive partner in the able-to-adapt and unable-to-adapt belief conditions. Right panel displays decision similarity (indexed by number of same choices on the object matching task) with the adaptive and unadaptive partner in the able-to-adapt and unable-to-adapt belief conditions. Error bars represent within-subject confidence intervals.

As in Experiment 1, to investigate the extent to which participants’ invested effort to charge their partners’ batteries (number of spacebar presses), we conducted a 2 × 2 mixed ANOVA with mean spacebar presses as a dependent variable, partner (adaptive and unadaptive) as a within-subjects factor, and belief (able-to-adapt and unable-to-adapt) as a between-subjects factor. The ANOVA revealed a significant main effect of partner, *F*(1,50) = 11.895, *p* < .001, η^2^ = .172, 95% CIs (within subject) [251.88, 282.12], [214.88, 245.12], but no significant main effect of belief, *F*(1,50) = .18, *p* = .67, η^2^ = .004, 95% CIs (within subject) [220.98, 257.02], [246.81, 269.19]. However, there was a significant interaction between partner and belief, *F*(1,50) = 7.188, *p* = .01, η^2^ = .104, 95% CIs (within subject) [253.98, 290.02], [187.98, 224.02], [250.81, 273.19], [242.81, 265.19] (see left panel of Figure 3), with participants persisting more to earn points for the adaptive partner than the unadaptive partner, but only in the partner able-to-adapt belief condition, *t*(25) = 3.609, *p* = .001, *d* = .708, and not in the partner unable-to-adapt belief condition, *t*(25) = .73, *p* = .474, *d* = .14. This result generalises our findings from Experiment 1, demonstrating that effortfully tailoring choices to our partner when trying to coordinate decisions can also foster commitment. Rather than just happening to have the same choices or preferences as someone, actively investing effort to align with the perspective of another agent leads a sense of commitment to arise.

Like in Experiment 1, we investigated how participants persisted throughout the experiment across the different conditions, to investigate whether participant’s motivation decreased at different rates. We carried out a LMM, with number of spacebar presses (per trial) as the response variable, partner, belief, and trial number as a fixed test effects, and participant number as a random effect. A likelihood ratio test between the full model and a null model containing only the random effects was significant, χ^2^ = 169.09, *p* < .001, meaning that our test effects explain a significant amount of the variance associated with the dependent variable. Our final model (found using a process of backward elimination) revealed a significant effect of partner, *t* = −4.51, *p* < .001, and no effect of belief, *t* = −1.30 *p* = .2, but an interaction between direction and knowledge, *t* = 3.31, *p* < .001. There was a main effect of trial, *t* = −12.49, *p* < .001, but no interaction between trial and direction or knowledge, demonstrating that although motivation did decrease throughout the experiment, it did so at the same rate for all conditions.

### Controlling for similarity

As in Experiment 1 with asynchrony, in this experiment the coordination accuracy was an index of similarity, because coordination success relies on the participant and partner making the same choices. On the accuracy data, we carried out a 2 × 2 mixed ANOVA, with mean coordination accuracy as a dependent variable, partner as a within-subjects factor and belief as a between-subjects factor. This analysis revealed a main effect of partner, *F*(1,50) = 47.21, *p* < .001, η^2^ = .48, 95% CIs (within subject) [70.2, 74.8], [61.2, 64.8], but no main effect of belief, *F*(1.50) = .056, *p* = .814, η^2^ = .001, 95% CIs (within subject) [65.7, 69.3], [65.8, 69.2]. There was also no interaction between partner and belief, *F*(1,50) = .99, *p* = .324, η^2^ = .01, 95% CIs (within subject) [71.2, 74.8], [69.3, 72.7], [60.2, 63.8], [62.3,65.7] (see right panel of Figure 3), suggesting that our manipulation did not lead to between group differences in decision-based similarity.

To control for overall similarity of decisions, we carried out an LMM, with number of spacebar presses (per trial) as the response variable, partner and belief as fixed test effects, coordination accuracy as a fixed control effect, and participant number as a random effect. A likelihood ratio test comparing the full model and a null model containing only the control and random effects was significant, χ^2^ = 8.56, *p* < .036, meaning that our test effects explain a significant amount of the variance associated with the dependent variable. Our final model (found using a process of backward elimination) revealed a significant effect of partner, *t* = −2.89, *p* = .004, but no effect of belief, *t* = −1.14, *p* = .256. There was also a significant main effect of coordination accuracy, *t* = 2.42, *p* = .015, demonstrating that coordination success does explain some of the variance associated with the dependent variable. However, the interaction between partner and belief was still significant, *t* = 2.72, *p* = .007, demonstrating that the difference in commitment which participants exhibited towards the two partners can cannot alone be explained by the differences in coordination success, thus can be attributed to our manipulation.

## General discussion

The current study aimed to investigate what it is about interpersonal coordination that leads people to develop a sense of commitment towards those with whom they interact. Across two experiments implementing two very different scenarios (action coordination and decision-making coordination), we demonstrate that an agent’s investment of effort to adapt movements or decisions to ensure successful and smooth coordination fosters a sense of commitment towards that agent.

Crucially, the effects of coordination upon commitment cannot be explained solely by inferences based on the similarity between the actors’ behaviour in a coordination context. Although for both experiments, we did observe a main effect of adaptivity (and therefore of movement similarity in Experiment 1 and of choice similarity in Experiment 2), the interaction between adaptivity and belief conditions reveals that the boost which coordination gave to commitment was driven by the inferences participants were led to draw about their partners’ willingness to effortfully adapt to the participant.

Nevertheless, it is noteworthy that we did observe that participants persisted more overall for the adaptive partner than the unadaptive partner in Experiment 1, regardless of the belief condition. Moreover, a main effect of overall decision similarity in our LMM analysis suggests that similarity of decisions may have played some role in Experiment 2. This suggests that both similarity and effort investment play a role in the emergence of commitment, perhaps to a different extent for timing similarity and decision similarity, or indeed through different mechanisms. Both experiments ensured that any similarity emerging from coordination was arbitrary (i.e., in timing or in decisions about meaningless stimuli) in the sense that this similarity did not inherently reflect anything socially meaningful. Rather, any socially meaningful perceptions of similarity between oneself and one’s partner would need to come from some process of drawing inferences on the basis of behavioural similarity. Accounts of this process that have been offered in the literature are either attributional, positing the attribution of similar goals or traits (Miles et al., 2009; Reddish et al., 2013), or low level, such as self-other merging or de-individuation (e.g., Cross et al., 2019; Hove & Risen, 2009). Perhaps, perceptual similarity in which there is a tight contingency between actions in real time is a more profound cue than more abstract decision-making, at least in our tasks. Alternatively, differences in the extent to which commitment is influenced by similarity or adaptation may be constrained by the prior expectations that the participant has of the task (Mills et al., 2019). For instance, participants may have been more sensitive to similarity in our temporal coordination task, simply because they had stronger expectations that their actions should be similar, compared with our decision-based coordination task.

Further research should compare how these different cues play out in the different coordination contexts (e.g., temporal similarity in decision-making and abstract similarity in action coordination). Moreover, examining the extent to which participants’ sensitivity to these cues depends on their prior expectations of the task may also help us to understand the mechanism by which coordination yields commitment, as well as other kinds of prosociality.

A further exploration of the motivational mechanisms by which coordination yields commitment is important not only to better understand our findings, but also potentially for a wider understanding of the factors working to sustain cooperation. What is it that drives a committed partner to persevere with a difficult task for the benefit of their adaptive partner, even though they would likely not interact with them in the future? We propose that the most plausible hypothesis is that an adaptive agent’s investment of effort creates a sense of debt or obligation towards that agent, leading to increased persistence to “repay one’s debt” to that agent (McGrath & Gerber, 2019). A sense of debt may drive increased commitment towards those who invest effort in a collaborative task, and lead those to not feel indebted therefore not commit to those who do not pull their weight in the interaction. This process may help us to cultivate long-term relationships with those whose effort demonstrates that they are diligent and reliable interaction partners and weed out those who are unreliable. This may also contribute to stable and consistent patterns of behaviour on a group level, with individuals willing to work hard for other members of their group, even when there is no clear reward for doing so.

There are several alternative hypotheses that may also be contributing to this effect. One alternative hypothesis is that, by investing the effort required to adapt, an agent signals that they are a considerate interaction partner, and that it is therefore worth cultivating a collaborative relationship with her. A second hypothesis is that an adaptive partner is perceived as more competent or intelligent than an unadaptive partner, and therefore more desirable as a future collaboration partner. Both of these two alternative hypotheses would lead us to expect that participants, if given the opportunity to choose their collaboration partner for a future task, would be more likely to choose an adaptive partner than an unadaptive partner. However, neither of these two hypotheses directly explains why participants in our experiments were particularly willing to persist to benefit partners who had demonstrated a willingness to adapt and invest effort (and with whom they knew they were not going to interact again). A further hypothesis is that participants are by default relatively committed to their partner, choosing to punish or withdraw commitment from those who do not pull their weight during their interaction. In our study, the absence of trial-by-trial feedback in the charging phase of our experiments means that withdrawal of commitment would have likely been out of spite rather than as a strategy to correct their partners’ behaviour (Jensen, 2010). Further research could investigate the extent to which punishment plays a role in the effects observed in this study, by examining trial-by-trial feedback about each other’s commitment behaviour, as well as how this behaviour influences adaptivity in the coordination task (i.e., repeated rounds with participants first completing one coordination trial and then one charging trial with feedback). Moreover, such a study could also investigate how punishing (or indeed rewarding) behaviour depends on participants’ default commitment behaviour (e.g., the extent to which they commit to someone they had not previously interacted with). Perhaps, those who have a tendency towards commitment are more likely to reward adaptive behaviour with commitment and punish unadaptive behaviour by withdrawing commitment.

Related to the above, one limitation of our study was that we failed to effectively investigate how prosocial attitudes, such as affiliation and empathy, are affected by effort investment in the form of adaptation, and how these attitudes may have had a moderating effect on commitment. Indeed, the focus that we put on investigating participants’ commitment in the face of an effortful and fatiguing task compromised our ability to effectively administer questionnaires to probe these attitudes. Further studies should investigate how the process by which coordination generates commitment is moderated by factors, such as affiliation and empathy, with a design that more carefully ensures that participants are motivated both to complete the commitment task but also to report their feelings towards their partners.

In addition to prosocial attitudes, further research should directly compare the influence that effort investment has on commitment with the influence that effort investment has on other kinds of prosocial behaviour (e.g., generosity as measured in the trust game). Such a study may simply reveal that other prosocial measures are also sensitive to effort investment as well as behavioural similarity. Alternatively, finding that other measures of prosociality lack the sensitivity to effort investment that we have observed with regards to commitment could yield important insights to the mechanisms by which commitments arise from joint actions. For example, compared with acting generously or kindly towards to someone, making a commitment to someone may entail a strong focus on reciprocity, with people only making commitments when they are sure that their partner would be likely to do the same for them.

A further potential line of research could aim to investigate the role that group membership plays in the effects that coordination has on commitment. There is evidence that the prosocial effects of coordination extend to outgroup members, and even lead to increased categorisation of outgroup members as ingroup (Atherton et al., 2019; Reddish et al., 2016). Reflection about the influence that group membership has upon the consequences of coordination for commitment may generate novel and testable predictions. For example, one prediction is that coordination fosters commitment in the same way that it fosters prosociality towards outgroups. Moreover, increased commitment to an outgroup member could be due to one re-categorising that person as an ingroup member. Alternatively, if the reason why coordination yields commitment is that it provides evidence that a partner may be a reliable co-actor in future interactions, then one may reserve this benefit for ingroup members, given that one is more likely to interact with ingroup members again, and more likely to compete for resources with outgroup members.

Interestingly, the current study demonstrates that, in addition to monetary incentives, effort is also an important currency for social interaction—in the same way that those who invest money for the public good are seen as trustworthy (Gächter et al., 2004), we show that those who invest effort may be seen as reliable and competent partners who are worth committing to. It would therefore be important to investigate to what extent findings from research using monetary incentives generalises to contexts in which the resource that is at stake is effort. Future research should explore how contributions of effort are monitored, compared and exchanged within cooperative activities (e.g., Ibbotson et al., 2019). For example, investigating how providing participants with reputational incentives may increase both the effort they are willing to invest into an interaction and the extent to which they are willing to make commitments.

In sum, our study contributes to the understanding of human social behaviour: both in our evolutionary past and today. Specifically, we demonstrate that commitment, a cornerstone of both small- and large-scale social interactions, emerges through the process of adapting to accommodate each other’s actions and decisions to coordinate. Adaptation not only facilitates successful coordination in the here and now—it also bolsters the commitment needed for successful future interactions. Our findings elucidate how, in addition to monetary rewards, effort investment in an interaction (e.g., adaptation) lay a foundation for short- and long-term cooperation. These insights are crucial to understanding why we humans endeavour to sustain cooperation even when we are in deep water and the tide is against us.

## Supplemental Material

sj-docx-1-qjp-10.1177_17470218221079830 – Supplemental material for The fruits of our labour: Interpersonal coordination generates commitment by signalling a willingness to adapt Click here for additional data file. Supplemental material, sj-docx-1-qjp-10.1177_17470218221079830 for The fruits of our labour: Interpersonal coordination generates commitment by signalling a willingness to adapt by Luke McEllin, Annalena Felber and John Michael in Quarterly Journal of Experimental Psychology
