# Supplementary material for: The fruits of our labour: Interpersonal coordination generates commitment by signalling a willingness to adapt
Source: Q J Exp Psychol (Hove). 2022 Mar 3;76(1):147–59. doi: 10.1177/17470218221079830 (PMC9773151; doi:10.1177/17470218221079830)
Supplement: sj-docx-1-qjp-10.1177_17470218221079830 – Supplemental material for The fruits of our labour: Interpersonal coordination generates commitment by signalling a willingness to adapt [file sj-docx-1-qjp-10.1177_17470218221079830.docx]

Supplementary Material for:

**The Fruits of our Labour: Interpersonal coordination generates commitment by signaling a willingness to adapt**

Luke McEllin, Annalena Felber, John Michael

**Supplementary Material 1: Battery charging screenshots**


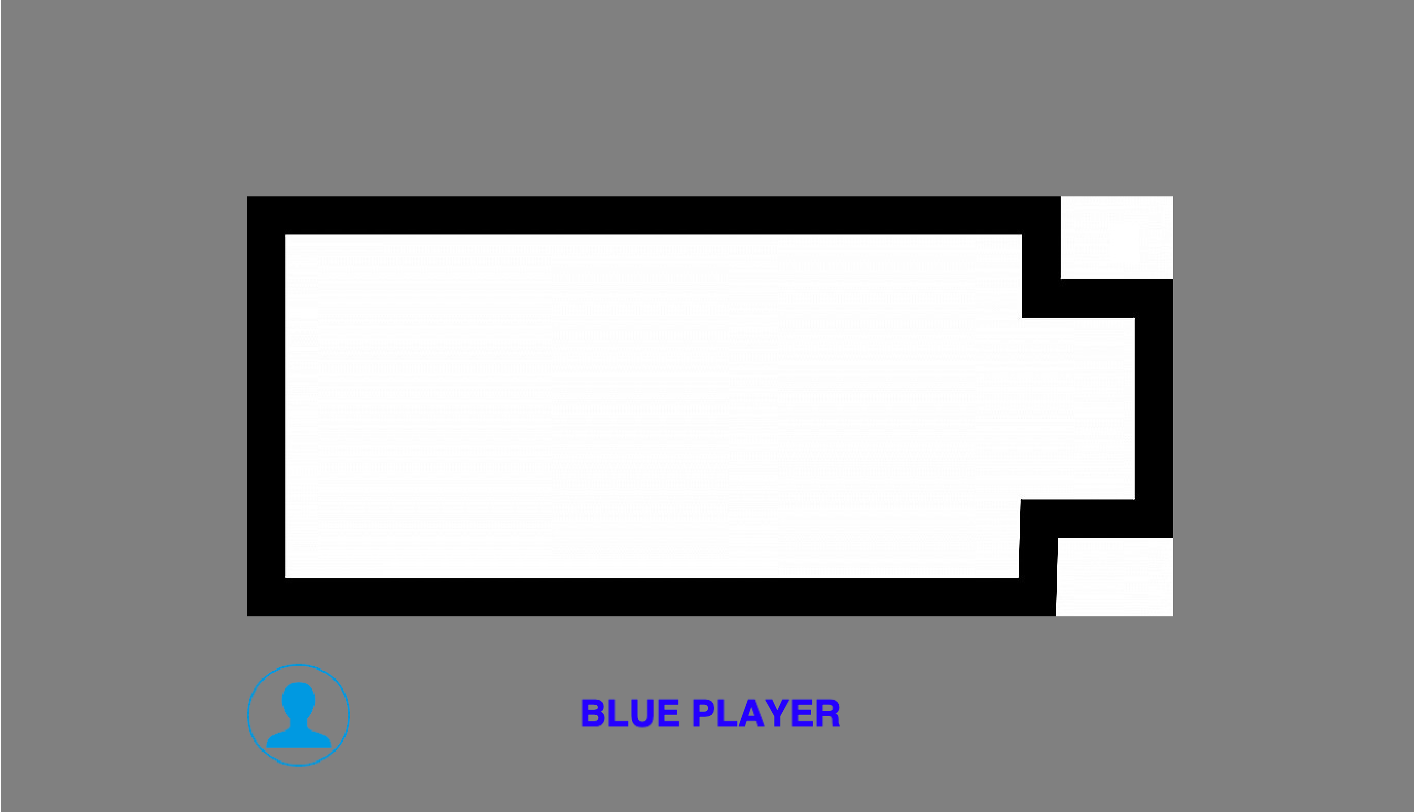


*Figure 1: Empty battery at start of the trial*


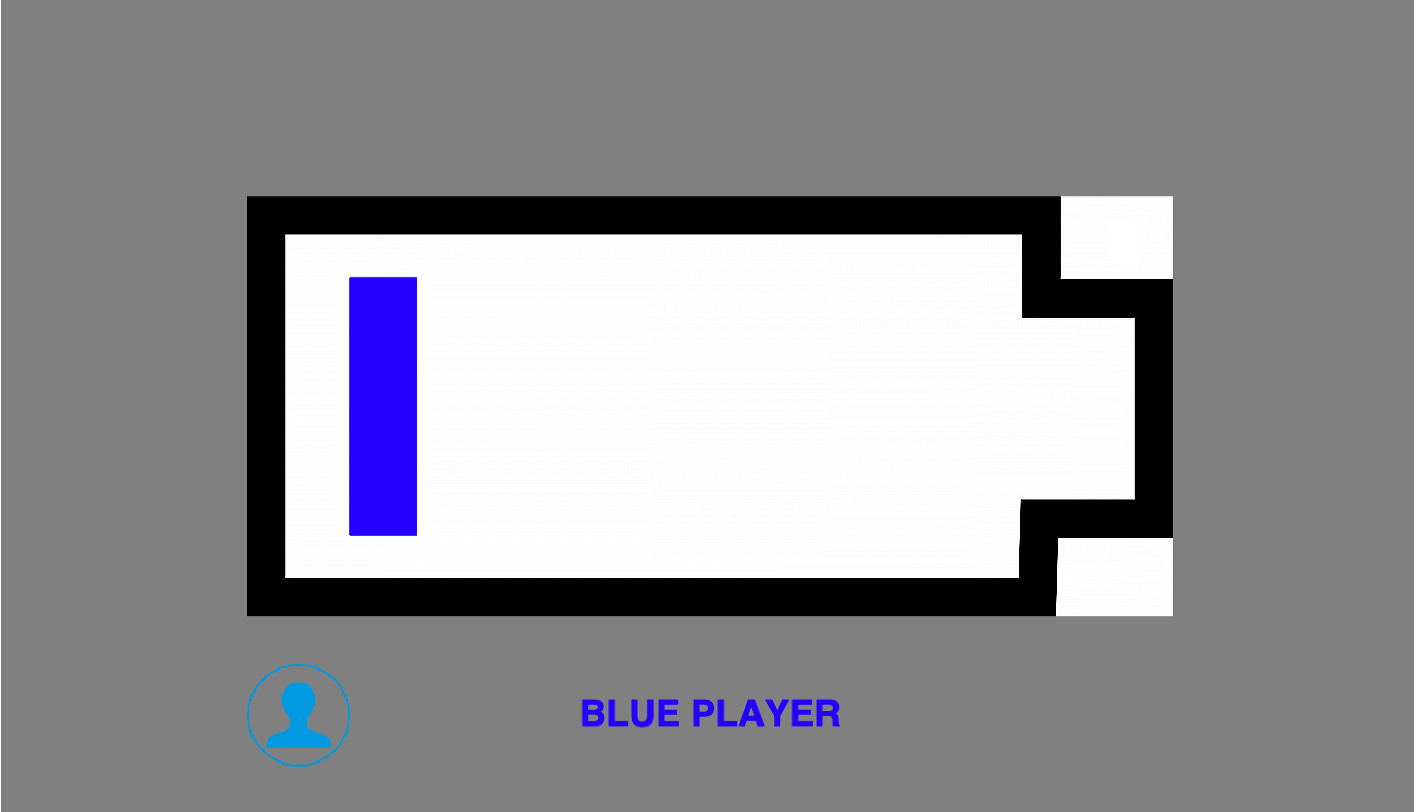


*Figure 2: Battery during early stages of charging*


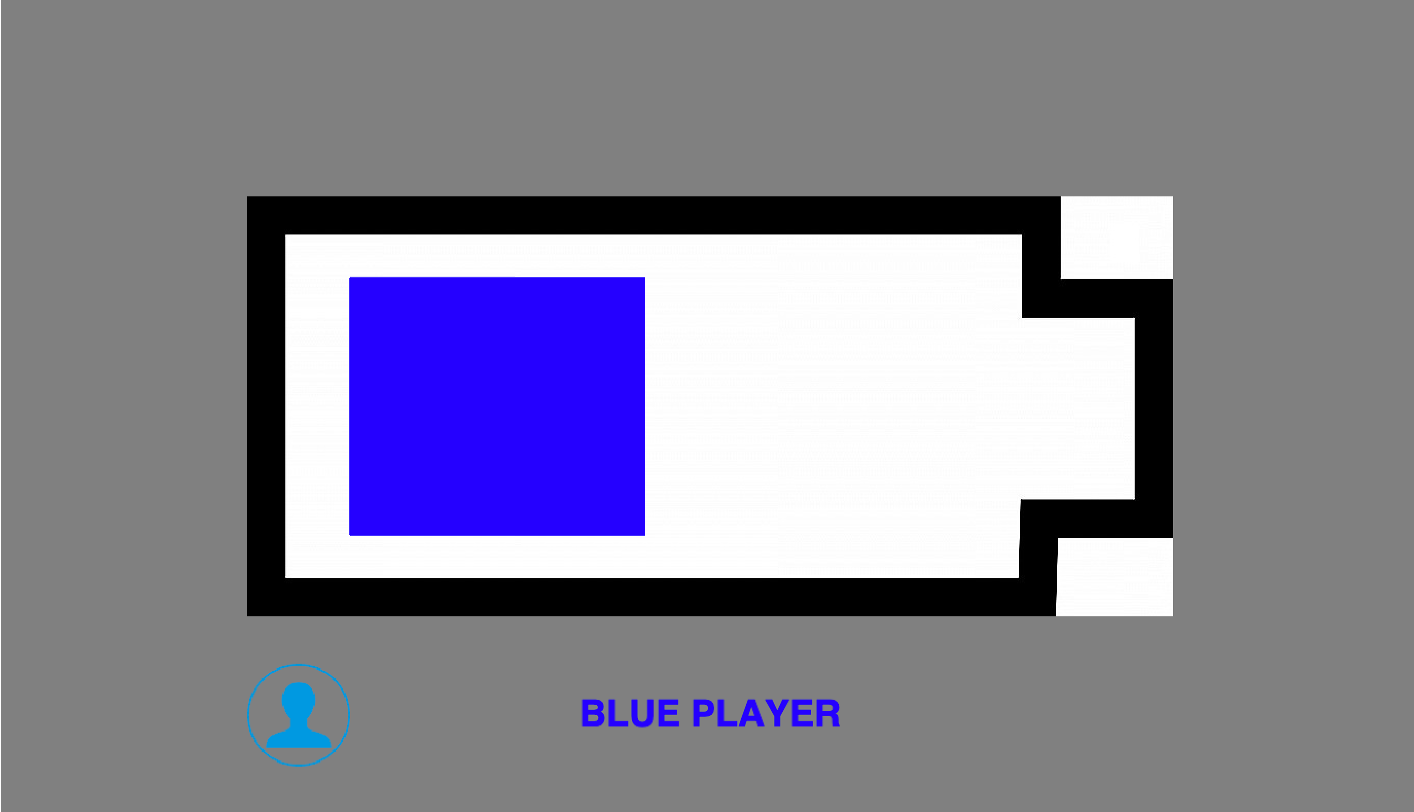


*Figure 3: Battery during middle stages of charging*


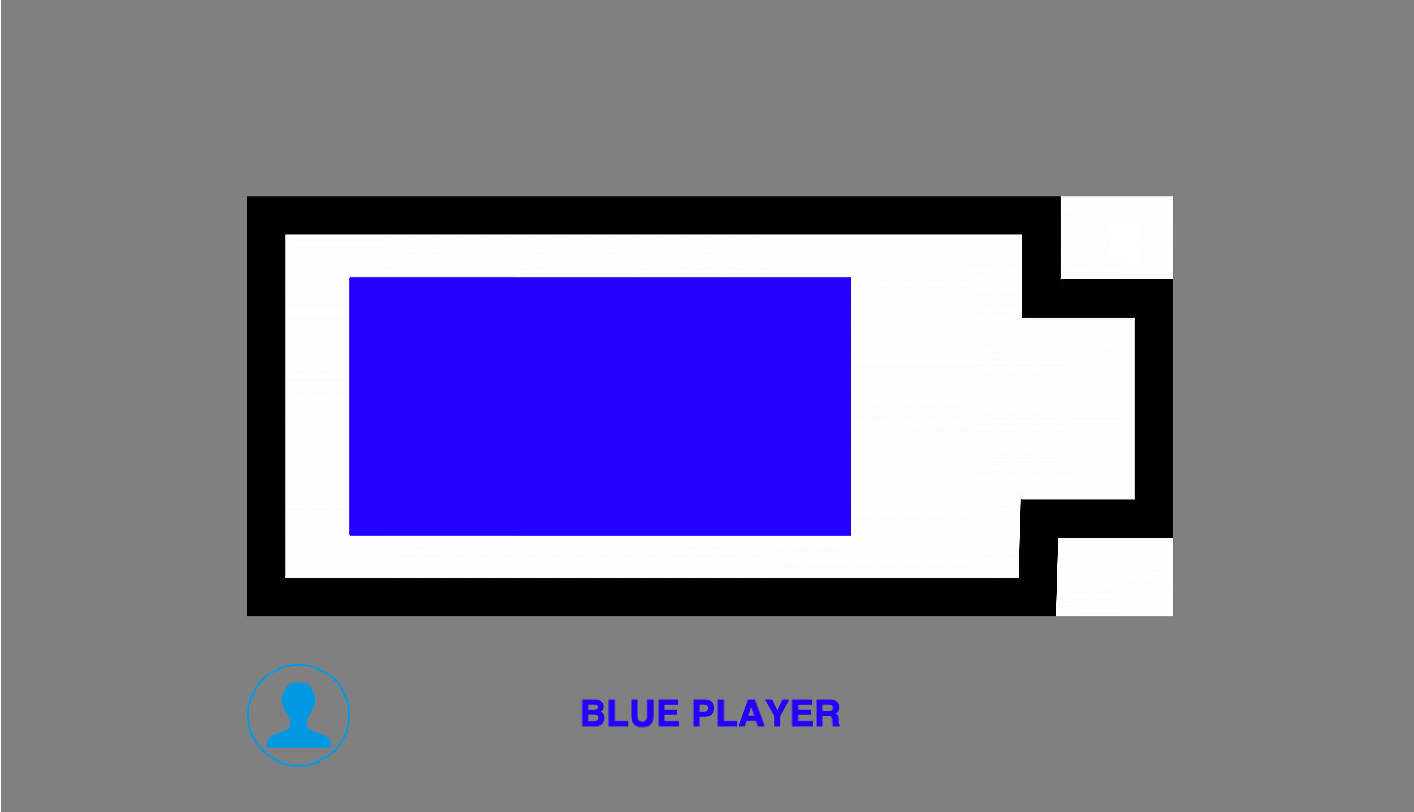


*Figure 4: Battery during later stages of charging*

**Supplementary Material 2: Schematic of decision-making coordination task**


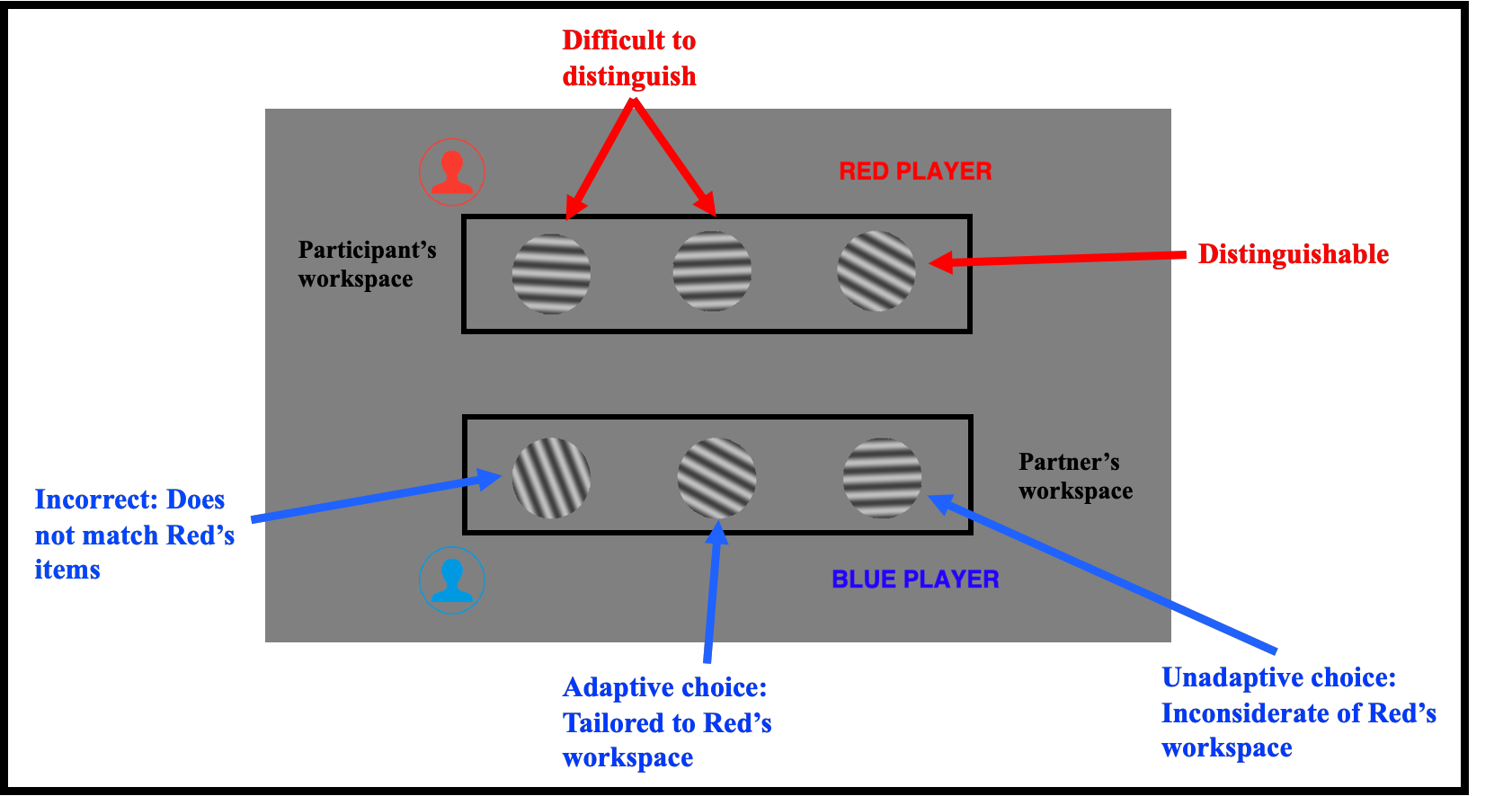


*Figure 1: Annotated screenshot of one decision-making coordination trial. Red text and arrows represent participant’s own choice. Blue text and arrows represent the partner’s choice from the participant’s point of view.*


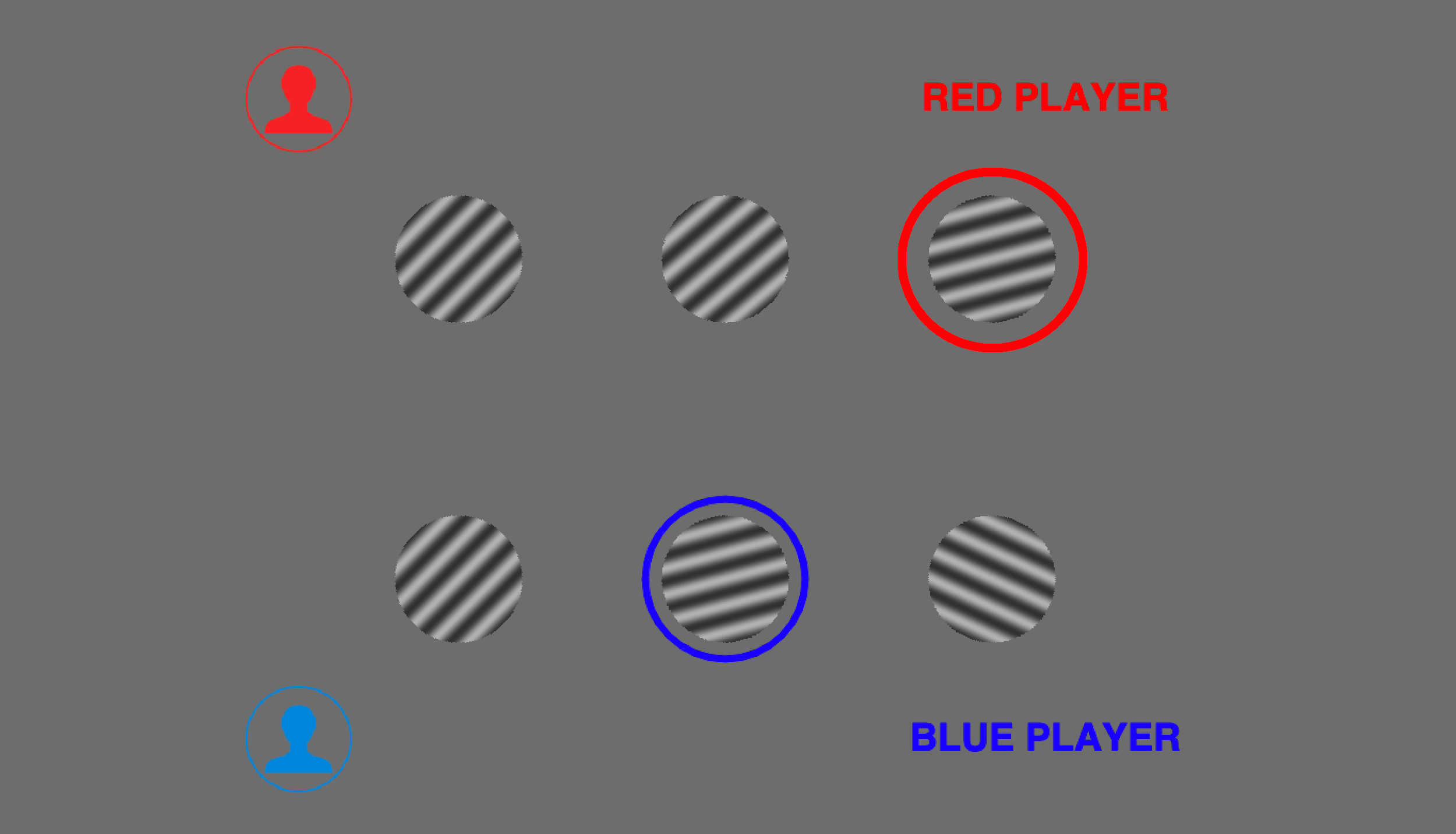


*Figure 2: Feedback after decision made. Colored circles represent choice of respective agent*

**Supplementary Material 3: Questionnaire data**

After excluding participants who did not complete any items on any of the questionnaires, we ended up with a sample of 23 participants in the Able-to-Adapt belief condition (5 did not complete) and 20 participants in the Unable-to-Adapt belief condition (8 did not complete). We averaged the affiliation items and the empathy items (see below for items used) into one composite score for affiliation and one composite score for empathy.

**Affiliation**

We excluded a further two participants, one from each of the Belief conditions, because they did not fill out any of the affiliation items (both scales were on separate pages so it is likely that they overlooked this). This left us with a final sample of 22 participants in the Able-to-Adapt condition, and 19 participants in the Unable-to-Adapt condition. Finally, we had 14 missing items (participants either did not answer, or answers were illegible), which we filled with the median score for that participant.

Because our data were not normally distributed (Shapiro-Wilk, p < .05), and because we had unequal sample sizes in our two groups, we decided to use Wilcoxon signed-rank tests (non-parametric equivalent to paired t-tests) in order to compare Affiliation ratings for Adaptive and Unadaptive partners for the Able-to-Adapt condition and the Unable-to-Adapt condition. For the Able-to-Adapt condition, participants rated Affiliation significantly higher with the Adaptive partner compared to the Unadaptive partner *z* = 132, *p* (bonferroni corrected) = .002, *r* = .94. For the Unable-to-Adapt condition, there was no significant difference between affiliation ratings for the Adaptive and Unadaptive partners, *z* = 56, *p* (bonferroni corrected) = .38, *r* = .43.


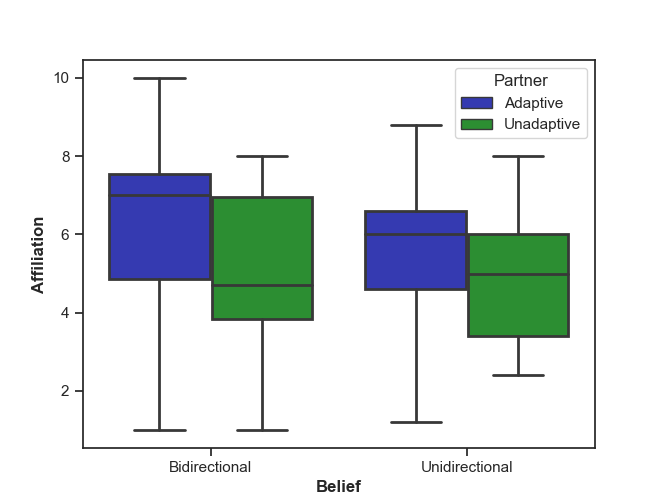


*Figure SM1: Box and whisker plots for Affiliation scores.*

**Empathy**

We excluded a further participant from the Able-to-Adapt condition due to failure to answer any of the empathy questions (both scales were on separate pages so it’s likely they overlooked this). This left us with a final sample of 22 participants in the Able-to-Adapt condition and 20 participants in the Unable-to-Adapt condition. Finally, we had 9 missing items (participants either did not answer, or answers were illegible), which we filled with the median score for that participant.

Because our data were not normally distributed (Shapiro-Wilk, p < .05), and because we had unequal sample sizes in our two groups, we decided to use Wilcoxon signed-rank tests in order to compare Empathy ratings for Adaptive and Unadaptive partners for the Able-to-Adapt condition and the Unable-to-Adapt condition. For the Able-to-Adapt condition, participants rated Affiliation significantly higher with the Adaptive partner compared to the Unadaptive partner *z* = 78, *p* (bonferroni corrected) = .003, *r* = .1. For the Unable-to-Adapt condition, there was no significant difference between affiliation ratings for the Adaptive and Unadaptive *z* = 51, *p* (bonferroni corrected) = 0.73, *r* = .31.

*
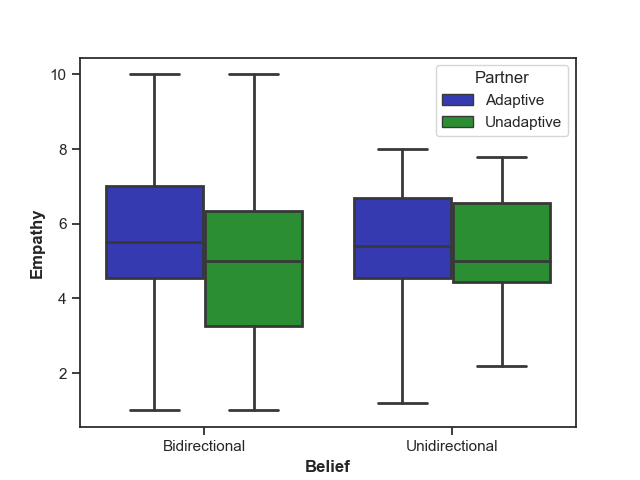
*

*Figure SM2: Box and whisker plots for Empathy scores.*

**﻿**

**Affiliation items**


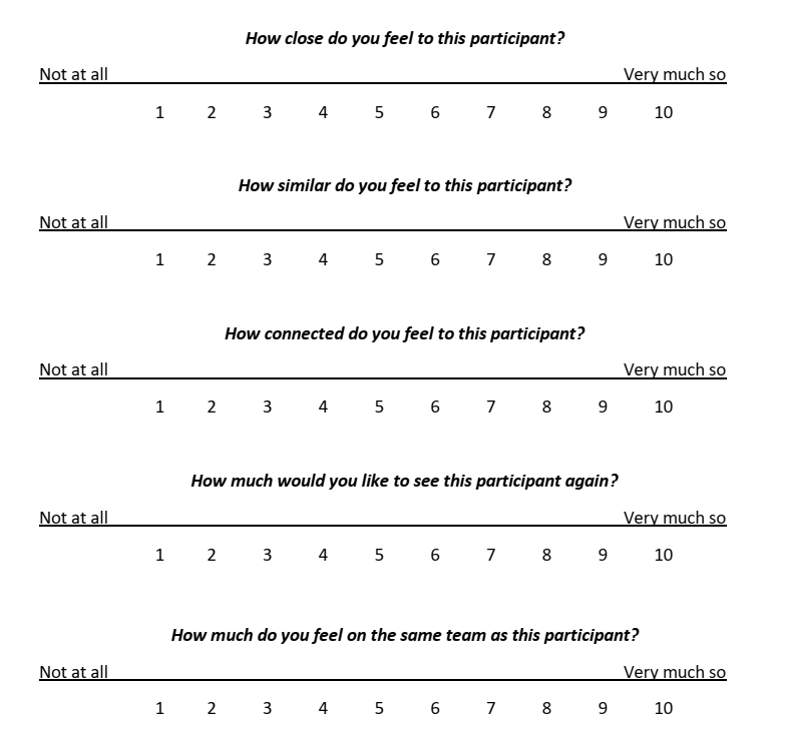


**Empathy items**

*
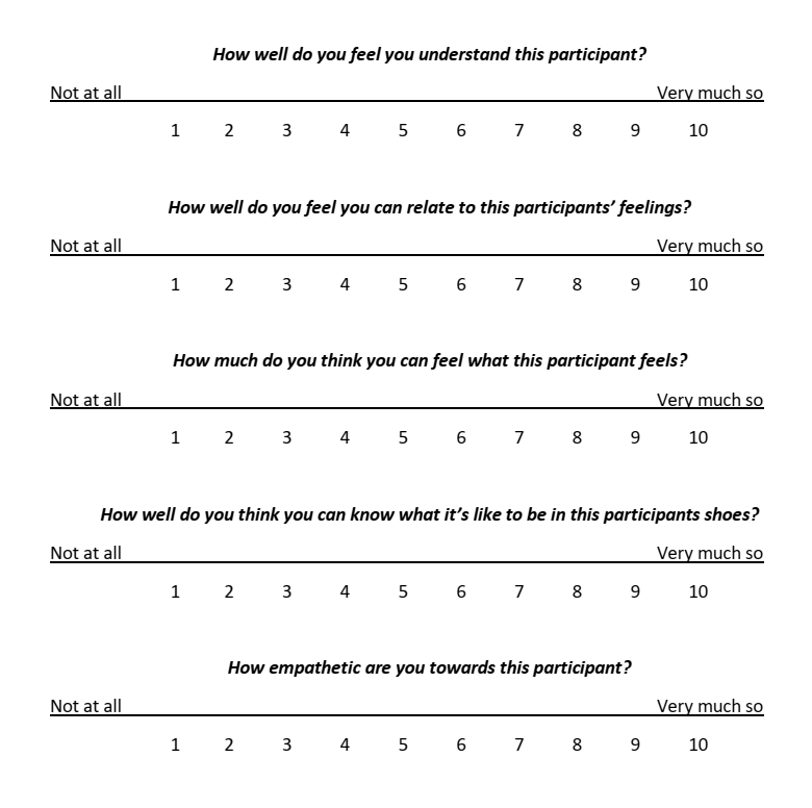
*

**Supplementary Material 4: Order effects**

Although the order in which participants interacted with the adaptive and unadaptive partner was counterbalanced, it may neverless be the case that we observe order effects in our study. For example, we may observe either more commitment towards the partner that the participant interacts with first, or we may find that participants commit more to the adaptive partner than the unadaptive partner, but only when they interact with the unadaptive partner first and the adaptive partner second (because this may make the adaptive partner seem even easier to interact after being frustrated with the unadaptive partner. Thus, we investigated order effects with a Linear Mixed Effects Model (LMM), with number of space presses (for each trial) as the response variable, and partner, belief, and order (whether the adaptive partner was first or second) as fixed test effects.

**Order effects: Experiment 1**

Our analysis revealed a main effect of partner, t = -3.14, p = .002, no effect of belief, t = -1.86, p = .06, and an interaction between partner and belief, t = 2.177, p = .029. There was no effect of order, t.= -1.63, p = .1, no interaction between partner and order, t = 1.1, p = .27, no interaction between belief and order, t = 1.54, p = .12. and no three-way interaction between partner, belief and order, t = -.98, p = .33, demonstrating that the order in which adaptive and unadaptive partners were presented had no effect on our measure of commitment.

**Order effects: Experiment 2**

Our analysis revealed a main effect of partner, t = -2.74, p = .008, no effect of belief, t = -0.96, p = .37, and an interaction between partner and belief, t = 2.06, p = .039. There was no effect of order, t.= -0.06, p = .95, no interaction between partner and order, t = -.29, p = .77, no interaction between belief and order, t = .04, p = .96. and no three-way interaction between partner, belief and order, t = -.15, p = .88, demonstrating that the order in which adaptive and unadaptive partners were presented had no effect on our measure of commitment.
